# Detecting schizophrenia at the level of the individual: relative diagnostic value of whole-brain images, connectome-wide functional connectivity and graph-based metrics

**DOI:** 10.1017/S0033291719001934

**Published:** 2020-08

**Authors:** Du Lei, Walter H. L. Pinaya, Therese van Amelsvoort, Machteld Marcelis, Gary Donohoe, David O. Mothersill, Aiden Corvin, Michael Gill, Sandra Vieira, Xiaoqi Huang, Su Lui, Cristina Scarpazza, Jonathan Young, Celso Arango, Edward Bullmore, Gong Qiyong, Philip McGuire, Andrea Mechelli

**Affiliations:** 1 Departments of Radiology, Huaxi MR Research Center (HMRRC), West China Hospital of Sichuan University, Chengdu, China; 2 Department of Psychosis Studies, Institute of Psychiatry, Psychology & Neuroscience, King's College London, De Crespigny Park, London, UK; 3 Center of Mathematics, Computation, and Cognition, Universidade Federal do ABC, Santo André, Brazil; 4 Department of Psychiatry and Neuropsychology, School of Mental Health and Neuroscience, Maastricht University Medical Center, Maastricht, The Netherland; 5 Mental Health Care Institute Eindhoven (GGzE), Eindhoven, The Netherlands; 6 School of Psychology & Center for neuroimaging and Cognitive genomics, NUI Galway University, Galway, Ireland; 7 Department of Psychiatry, School of Medicine, Trinity College Dublin, Dublin, Ireland; 8 Department of General Psychology, University of Padua, Padua, Italy; 9 IXICO plc, London, UK; 10 Hospital General Universitario Gregorio Marañon. School of Medicine, Universidad Complutense Madrid. IiSGM, CIBERSAM, Madrid, Spain; 11 Brain Mapping Unit, Department of Psychiatry, University of Cambridge, Cambridge, UK; 12 Psychoradiology Research Unit of the Chinese Academy of Medical Sciences (2018RU011), West China Hospital of Sichuan University, Chengdu, Sichuan, China

**Keywords:** functional connectivity, graph theoretical analysis, machine learning, neuroimaging, schizophrenia.

## Abstract

**Background:**

Previous studies using resting-state functional neuroimaging have revealed alterations in whole-brain images, connectome-wide functional connectivity and graph-based metrics in groups of patients with schizophrenia relative to groups of healthy controls. However, it is unclear which of these measures best captures the neural correlates of this disorder at the level of the individual patient.

**Methods:**

Here we investigated the relative diagnostic value of these measures. A total of 295 patients with schizophrenia and 452 healthy controls were investigated using resting-state functional Magnetic Resonance Imaging at five research centres. Connectome-wide functional networks were constructed by thresholding correlation matrices of 90 brain regions, and their topological properties were analyzed using graph theory-based methods. Single-subject classification was performed using three machine learning (ML) approaches associated with varying degrees of complexity and abstraction, namely logistic regression, support vector machine and deep learning technology.

**Results:**

Connectome-wide functional connectivity allowed single-subject classification of patients and controls with higher accuracy (average: 81%) than both whole-brain images (average: 53%) and graph-based metrics (average: 69%). Classification based on connectome-wide functional connectivity was driven by a distributed bilateral network including the thalamus and temporal regions.

**Conclusion:**

These results were replicated across the three employed ML approaches. Connectome-wide functional connectivity permits differentiation of patients with schizophrenia from healthy controls at single-subject level with greater accuracy; this pattern of results is consistent with the ‘dysconnectivity hypothesis’ of schizophrenia, which states that the neural basis of the disorder is best understood in terms of system-level functional connectivity alterations.

## Introduction

Schizophrenia is a potentially severe psychiatric disorder, characterized by delusions, hallucinations, and disorganized thinking (Hu *et al*., 2017), which affects 2–3% of the world's population (Rajji *et al*., 2014; Nowak *et al*., 2016). The aetiology and neuropathology of this complex disorder are not well understood, and an accurate diagnosis can be slow due to the lack of reliable biomarkers. When neuroimaging became widely available two decades ago, it was hoped that this would lead to the development of imaging-based biomarkers that could be used to inform diagnostic and prognostic assessment of individual patients. The development of such biomarkers, however, has proved elusive due to the presence of complex, distributed, and subtle alterations that vary from one individual to another depending on their unique clinical profile. Traditional analytical techniques, which provide average estimates at the group level, have proved inadequate for detecting such alterations and dealing with the high degree of clinical heterogeneity amongst patients. In order to address this challenge, in the past decade, the neuroimaging community has begun to make use of an alternative analytic approach known as machine learning (ML). The advantage of ML relative to traditional analytical techniques is two-fold: firstly, ML methods are multivariate and therefore take the inter-correlation between input variables (e.g. voxels) into account; secondly, ML methods allow inferences at the individual rather than group level, and therefore generate results with greater potential of being translated into clinical tests.

Several studies have applied ML to neuroimaging data acquired from people at various stages of psychiatric illness with varying degree of success (Orrù *et al*., 2012; Pettersson-Yeo *et al*., 2013; Kim *et al*., 2016, ). The vast majority of studies have used whole-brain images as input for single-subject classification without performing any *a priori* feature extraction (Lueken *et al*., 2015; Rehme *et al*., 2015). However, the human brain is a highly interconnected network, and the emergence of psychiatric illness is thought to be underpinned by a disruption of normal functional integration amongst cortical and subcortical regions. Therefore, a number of studies have employed a second approach that involves estimating functional integration across the whole brain and using this as input for single-subject classification (Shen *et al*., 2010; Iidaka, 2015). In addition, following recent advances in graph-based theoretical analysis, it is now possible to estimate the topological properties of the human brain in health and disease (Bullmore and Sporns, 2009; Bullmore and Bassett, 2011). This has revealed that the human brain has a small-world organization (characterized by a high local specialization and a high global integration between the brain regions) (Salvador *et al*., 2005; He *et al*., 2007), and that these networks are anatomically and functionally disrupted in individuals with psychiatric diseases (Lynall *et al*., 2010; Pettersson-Yeo *et al*., 2011). Several recent studies, therefore, have employed a third approach that involves using graph-based analytic methods to estimate the topological properties of the brain in patients and then using this information as input for single-subject classification (Cheng *et al*., 2015; Khazaee *et al*., 2016).

Because none of the existing studies on schizophrenia has employed all three approaches, at present, it is unclear which type of alteration best captures the neural correlates of the disorder at the level of the individual patient. This information would be important both from a clinical translation and theoretical modelling perspective. From a clinical translation perspective, understanding which type of approach is most discriminant between patients and controls would help us develop more accurate ML algorithms. From a theoretical modelling perspective, such understanding would shed light on the neural correlates of schizophrenia and inform current neurobiological models of the illness.

The present article, therefore, aims to elucidate the neural correlates of schizophrenia by comparing the diagnostic accuracy of three distinct metrics of brain function: pre-processed whole-brain images, connectome-wide functional connectivity and graph-based metrics. In order to assess the reliability of the findings, we perform this comparison using three ML methods of varying degrees of complexity and abstraction: logistic regression (LR) (lower complexity), support vector machine (SVM) (medium complexity) and deep learning (DL) technology (higher complexity). We used resting-state functional magnetic resonance imaging (rs-fMRI) data acquired at five different sites from a total of 295 patients with schizophrenia and 452 healthy controls. Our main dependent variable of interest was the accuracy of classification for the comparison between patients and controls. In light of the current understanding of psychiatric disorders as abnormalities in connectome-wide functional connectivity (Rubinov and Bullmore, 2013) and topological properties (Suo *et al*., 2018), we hypothesized that (i) the use of measures of functional integration (i.e. connectome-wide functional connectivity and graph-based metrics) would allow diagnostic classification with higher level of accuracy than whole-brain images. In addition, given that whole-brain functional integration may provide a richer characterization of network-level functioning than topological properties, we hypothesized that (ii) the use of connectome-wide matrices would allow a higher level of accuracy than graph-based analytic metrics. Finally, based on a previous large-scale investigation (Sabuncu *et al*., 2015), we hypothesized that (iii) these findings would be consistent across different ML methods including logistic regression, SVM and DL technology, thereby supporting the reliability of our conclusions.

## Materials and methods

### Participants

We used five datasets, each including subjects with a diagnosis of schizophrenia and healthy controls. For information on participants in each dataset, see online Supplemental Information.

The combination of the five datasets yielded 295 patients with schizophrenia and 452 healthy controls. The demographic and clinical characteristics of the participants are presented in Table 1.

**Table 1.** Demographic and clinical characteristics of participants^a^

	Dataset 1		Dataset 2		Dataset 3		Dataset 4		Dataset 5	
	SCZ	CON	SCZ	CON	SCZ	CON	SCZ	CON	SCZ	CON
Sample size	68	72	56	132	49	63	32	83	90	102
Disease stage	EST^b^	–	EST	–	EST	–	EST	–	FE	–
Age (years)	38.10 ± 14.13	35.87 ± 11.74	36.16 ± 8.52	30.99 ± 8.62	29.02 ± 6.39	29.60 ± 10.59	40.94 ± 10.90	28.09 ± 8.98	24.31 ± 7.77	30.56 ± 15.21
Gender (M/F)	55/13	51/21	42/14	69/63	38/11	25/38	24/8	38/45	33/57	49/53
Handedness (R/L/B)	56/10/2	69/1/2	NA	NA	40/7/2	54/7/2	32/0/0	83/0/0	90/0/0	102/0/0
Education (years)	NA	NA	NA	NA	16.61 ± 1.99	17.38 ± 2.00	14.72 ± 4.41	17.73 ± 3.28	12.13 ± 3.21	12.27 ± 3.18
Medication (An/Dn)	68/0	NA	45/4^c^	NA	48/1	NA	23/5^d^	NA	0/90	NA
PANSS total	58.78 ± 14.35^e^	NA	NA	NA	44.16 ± 12.40	NA	NA	NA	96.46 ± 17.02^f^	NA
PANSS positive	14.36 ± 4.78^e^	NA	NA	NA	10.10 ± 4.43	NA	NA	NA	26.17 ± 5.40^f^	NA
PANSS negative	15.00 ± 5.36^e^	NA	NA	NA	10.81 ± 5.29	NA	NA	NA	17.90 ± 7.18^f^	NA
PANSS general	29.42 ± 8.55^e^	NA	NA	NA	23.24 ± 5.49	NA	NA	NA	48.05 ± 9.09^f^	NA
SAPS	NA	NA	23.16 ± 17.00^g^	NA	NA	NA	7.58 ± 12.28^h^	NA	NA	NA
SANS	NA	NA	28.30 ± 16.14^g^	NA	NA	NA	13.33 ± 17.85^h^	NA	NA	NA

a Data are presented as mean±standard deviation.

b Patients were diagnosed with established schizophrenia if duration of illness was more than 24 months.

c Data available for 49 of 56 patients.

d Data available for 28 of 32 patients.

e Data available for 50 of 68 patients.

f Data available for 88 of 90 patients.

g Data available for 50 of 56 patients.

h Data available for 24 of 32 patients.

SCZ, schizophrenia; CON, control; EST, established; FE, first episode; PANSS, Positive and Negative Syndrome Scale; SAPS, Scale for the Assessment of Positive Symptoms; SANS, Scale for the Assessment of Negative Symptoms; M, male; F, female; R, right; L, left; B, ambidextrous; An, antipsychotic medication; Dn, drug-naïve; NA, not available.

### MRI acquisition

At each site, the rs-fMRI images were acquired by the Echo-Planar Imaging (EPI) sequence. For information on MRI acquisition for each dataset, see online Supplemental Information.

### Data pre-processing

Image pre-processing was performed using SPM8 (http://www.fil.ion.ucl.ac.uk/spm). The first 10 volumes were discarded to minimize the impact of the instability in the initial MRI signal. The remaining images were corrected for intra-volume acquisition time delay and inter-volume geometric displacement of head motion. None of the subjects included in the present investigation showed excessive head motion during scanning (defined as translational movement >1.5 mm and/or rotation >1.5°). After these corrections, the images were spatially normalized to a 3 × 3 × 3 mm^3^ Montreal Neurological Institute (MNI) 152 template and then linearly detrended and temporally bandpass filtered (0.01–0.08 Hz) to remove low-frequency drift and high-frequency physiological noise. Finally, the global signal, the white matter signal, the cerebrospinal fluid (CSF) signal and the motion parameters were regressed out (Fox *et al*., 2009). The resulting images were then used as input features for the subsequent ML analyses, in order to examine the diagnostic value of pre-processed whole-brain images.

### Network construction

The graph theoretical network construction was performed using GRETNA software (http://www.nitrc.org/projects/gretna/) (Wang *et al*., 2015). First, to define the brain nodes, the whole brain was divided into 90 cortical and subcortical regions of interest – each representing a network node – using the automated anatomical labelling atlas. Next, to define the edges of the network, we extracted the mean time series of each region, and calculated Pearson's correlations of the mean time series between all pairs of nodes. This process resulted in a 90 × 90 weighted correlation matrix for each subject. This correlation matrix was then used as input feature for the subsequent ML analyses, in order to examine the diagnostic value of connectome-wide functional connectivity.

### Network analysis and graph-based metrics extraction

To address the problem that networks of different subjects differed in the number of edges (Wen *et al*., 2011), we applied a range of sparsity thresholds, *S*, to the correlation matrices consistent with previous studies (Lei *et al*., 2015; Suo *et al*., 2015). Here *S* was defined as the ratio of the number of existing edges divided by the maximum possible number of edges in a network. This thresholding strategy results in each graph having the same number of edges, thereby enabling the investigation of between group differences in relative network organization (Zhang *et al*., 2011). Here the use of a range of sparsity thresholds *S* generated a threshold range of 0.10 < *S* < 0.34 with an interval of 0.01.

For each sparsity level, we calculated both global and nodal network metrics. The global metrics were of two kinds: (i) small-world parameters [for definitions see (Watts and Strogatz, 1998)] including the clustering coefficient *C*_p_, characteristic path length *L*_p_ [calculated as the harmonic mean distance between all possible pairs of regions to address the disconnected graphs dilemma (Newman, 2003)], normalized clustering coefficient *γ*, normalized characteristic path length *λ*, and small-worldness *σ*; and (ii) network efficiency parameters [for definitions see (Latora and Marchiori, 2001)] including the local efficiency *E*_loc_ and global efficiency *E*_glob_. The nodal centrality metrics were the nodal degree, nodal efficiency, and nodal betweenness. Finally, for each network metric, we calculated the area under the curve (AUC) over the sparsity range from *S*_1_ to *S*_*n*_ with an interval of *ΔS*, where *S*_1_ = 0.10, *S*_*n*_ = 0.34 and *ΔS* = 0.01. The AUC provides a summarized scalar for the topological characterization of brain networks that is independent of a single threshold selection and sensitive to topological alterations in brain disorders (Zhang *et al*., 2011). The AUC values for the above global and nodal metrics were then used as input features for the subsequent ML analyses, in order to examine the diagnostic value of graph-based metrics.

### Controlling for age and gender effects

For each measure of brain function (i.e. whole-brain images, connectome-wide matrices or graph-based metrics) in each dataset, we build a regression model that represented how the measure varied with age and gender in the control sample, and then subtracted age- and gender-related variance from the actual measures. This was done using the Gaussian process regression method and kernel function that were used in a previous investigation (Kostro *et al*., 2014), with the regression model based on control subjects only. This ensured that the residuals represented variation due to disease-related effects only, and could not be explained by age or gender effects. These residuals were then used as features for the ML analyses.

### ML models

In the present study, we used three ML methods – LR, SVM and DL to perform single-subject classification. These methods were chosen in light of their widespread use amongst the neuroimaging community and their varying degrees of complexity and abstraction. In particular, LR is considered to be the ‘simplest’ ML method; SVM is associated with medium levels of complexity and abstraction, and is the most widely used ML method in psychiatric neuroimaging; and DL is thought to have the greatest potential of detecting hidden patterns in the data, due to its higher levels of complexity and abstraction. These methods are described in detail in online Supplemental Information.

### Measuring the performance of ML models

**Fig. 1.**
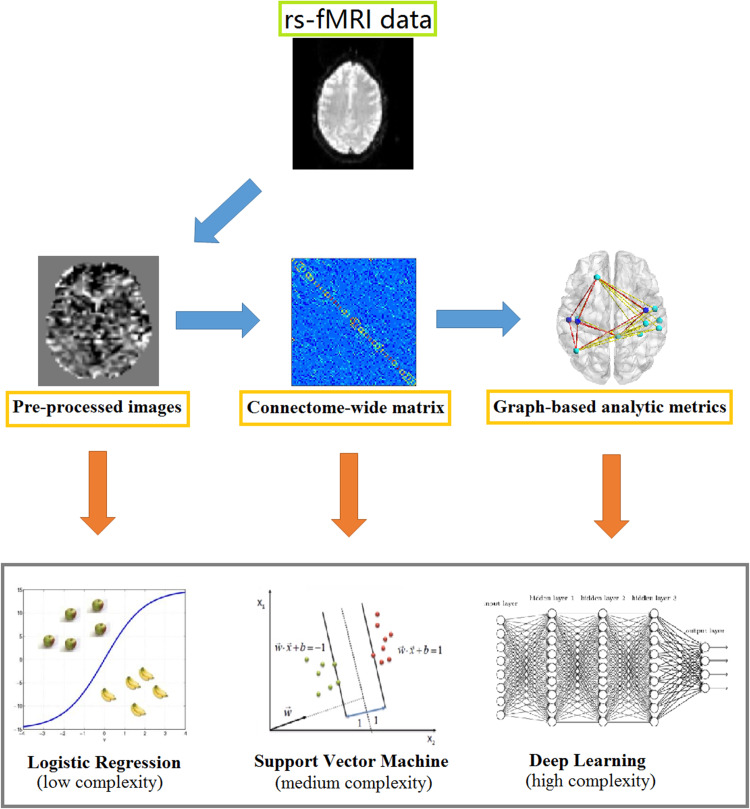
Overview of the employed classification approach showing the main steps in the pipeline.

In order to determine which type of input data would allow the highest accuracy of classification, we applied the three ML approaches (i.e. LR, SVM and DL) to the whole-brain normalized images (4D volume with time as the fourth dimension), the connectome-wide matrices (90 × 90 Pearson correlation matrix) and the graph-based analytic metrics (i.e. seven global metrics *C*_p_, *L*_p_ *γ*, *h λ*, *σ*; *E*_loc_, *E*_glob_ and 270 nodal metrics including nodal degree, nodal efficiency, and nodal betweenness × 90 brain regions). For each type of data and each type of ML method, a stratified 5-folds cross-validation was performed to measure the mean balanced accuracy of single-subject classification. This involved dividing the entire dataset into five folds that preserved the relative proportion of the two classes, and then using four folds as training set and the remaining fold as test set. To estimate the significance for each ML model and data features, we performed a nonparametric permutation test to calculate a *p* value for the balanced accuracy (Golland and Fischl, 2003). This involved repeating the classification procedure 1000 times with different random permutations of the group labels. We then counted the number of times the balanced accuracy was higher for the permuted labels than the real labels, and divided this number by 1000 to calculate a *p* value. Figure 1 shows an overview of the employed classification approach showing the main steps in the pipeline.

## Results

### Classification performance

The results of the single-subject classification of patients and healthy controls, including accuracies, sensitivities, specificities, and *p* values, are reported in Table 2. It can be seen that when using whole-brain normalized images data, the mean balanced accuracy of classification was at chance level; in particular, the average balanced accuracy of classification across the five datasets was about 53% for all three ML methods.

**Table 2.** Classification of patients with schizophrenia and healthy controls^a^

	Whole-brain images			Functional connectivity			Graph-based metrics		
	LR (%)	SVM (%)	DL (%)	LR (%)	SVM (%)	DL (%)	LR (%)	SVM (%)	DL (%)
Dataset 1									
Accuracy	53.54	59.47	50.27	81.81	83.33	84.05	72.28	74.99	69.07
Sensitivity	38.13	52.75	32.64	69.23	100.00	73.63	58.46	62.75	66.04
Specificity	68.95	66.19	67.90	94.38	66.67	94.48	86.10	87.24	72.10
*p* value^b^	0.155	0.056	0.421	<0.001	<0.001	<0.001	<0.001	<0.001	<0.001
Dataset 2									
Accuracy	50.00	50.00	50.00	80.01	77.14	77.97	65.28	71.21	68.84
Sensitivity	40.00	40.00	40.00	75.15	87.58	62.73	53.33	53.79	55.15
Specificity	60.00	60.00	60.00	84.87	66.70	93.22	77.24	88.63	82.54
*p* value^b^	0.375	0.433	0.648	<0.001	<0.001	<0.001	<0.001	<0.001	<0.001
Dataset 3									
Accuracy	53.05	54.65	51.44	82.41	87.31	80.50	79.66	78.69	72.74
Sensitivity	58.67	58.67	42.89	71.11	100.00	75.11	73.56	65.33	65.33
Specificity	47.44	50.64	60.00	93.72	74.62	85.90	85.77	92.05	79.62
*p* value^b^	0.180	0.088	0.777	<0.001	<0.001	<0.001	<0.001	<0.001	<0.001
Dataset 4									
Accuracy	51.10	54.37	53.74	81.19	79.62	81.69	66.82	63.81	65.00
Sensitivity	60.00	62.86	62.86	80.48	96.67	68.10	58.57	46.67	41.90
Specificity	42.21	45.88	44.63	81.91	62.57	95.29	75.07	80.96	88.09
*p* value^b^	0.408	0.296	0.3182	<0.001	<0.001	<0.001	<0.001	<0.001	<0.001
Dataset 5									
Accuracy	56.00	56.00	54.52	79.44	81.32	80.96	57.19	71.33	67.41
Sensitivity	14.52	60.00	52.22	67.78	97.78	67.78	46.67	46.67	61.11
Specificity	86.67	52.00	56.81	91.10	64.86	94.14	67.71	96.00	73.71
*p* value^b^	0.236	0.087	0.196	<0.001	<0.001	<0.001	0.019	<0.001	<0.001
Average Sensitivity	42.26	54.86	46.12	72.75	96.41	69.47	58.12	55.04	57.91
Average Specificity	61.05	54.94	57.87	89.20	67.08	92.61	78.38	88.98	79.21
Average Accuracy	52.74	54.90	51.99	80.97	81.74	81.03	68.25	72.00	68.61

a Sensitivity and specificity were computed considering the patient group as the positive class.

b Statistical significance was estimated using the permutation method (1000 permutations).

SVM, support vector machine; LR, logistic regression; DL, deep learning

When using graph-based analytic metrics, the mean balanced accuracy of classification across the five datasets was around 69% (68.25%, 72.00%, and 68.61% for LR SVM, and DL, respectively).

In contrast, using connectome-wide matrices, it was possible to discriminate between patients and controls at single-subject level with above chance mean balanced accuracy; in particular, the average balanced accuracy of classification across the five datasets was 80.97%, 81.74%, and 81.03% for LR SVM, and DL, respectively.

This pattern of results suggests that (i) connectome-wide matrices provide superior balanced accuracy of classification between patients and controls relative to graph-based analytic metrics; and (ii) the superiority of connectome-wide matrices is expressed across a range of ML method with varying degrees of complexity and abstraction.

### Which regions provided the greatest contribution to single-subject classification?

**Fig. 2.**
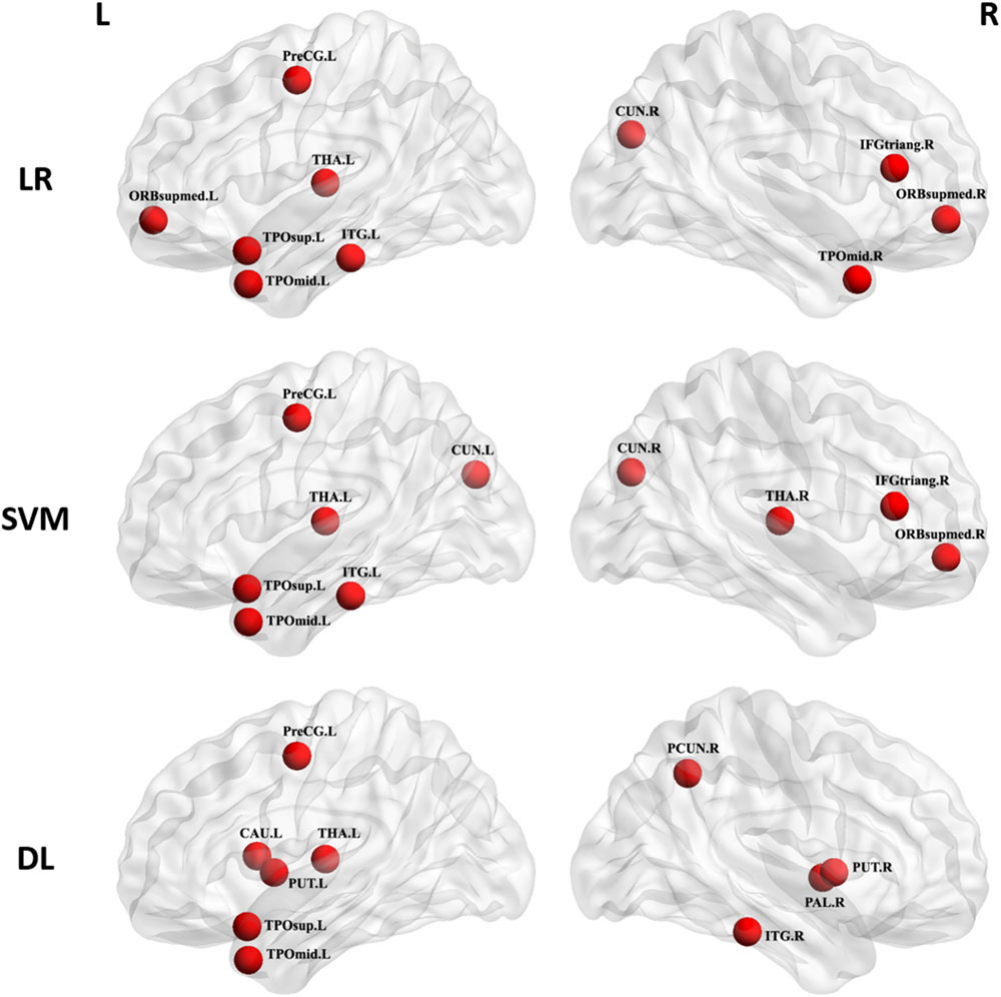
Regions providing the greatest contribution to single-subject classification of patients and controls across the five datasets. The nodes were mapped onto the cortical surfaces by using the BrainNet Viewer package (http://www.nitrc.org/projects/bnv). CAU, Caudate nucleus; CUN, Cuneus; IFGtriang, inferior frontal gyrus, triangular part; ITG, Inferior temporal gyrus; ORBsupmed, Superior frontal gyrus, medial orbital part; PAL, Pallidum; PCUN, Precuneus; PreCG, Precentral gyrus; PUT, putamen; TPOmid, Temporal pole: middle temporal gyrus; TPOsup, Temporal pole: superior temporal gyrus; THA, thalamus; R, right hemisphere; L, left hemisphere.

Having identified connectome-wide functional connectivity as the most powerful measure for capturing neurophysiological alternations in schizophrenia, we proceeded to examine the regions contributing to its superior performance. In the LR and SVM models, we computed the mean absolute value of the weights of the model across the different folds of the cross-validation, while in the DL models we used the Kim's approach (Kim *et al*., 2016) where they used a linear combination of the weights across layers. For each region, the mean value of the weights for its functional connectivity with the remaining 89 regions was calculated. The 10 brain regions with the highest mean values, computed by averaging the weights across the five datasets, are reported in Table 3 and represented in Fig. 2. It can be seen that the inferior temporal gyrus, the temporal pole, the precentral gyrus and the thalamus featured consistently across the different ML methods. In contrast, other regions (e.g. putamen) were detected in some cases (e.g. DL) but not others (e.g. LR and SVM).

**Table 3.** Top 10 most relevant brain regions for the classification analysis

LR	SVM	DL
Inferior temporal gyrus R	Thalamus L	Temporal pole: middle temporal gyrus L
Thalamus L	Cuneus R	Inferior temporal gyrus R
Temporal pole: superior temporal gyrus L	Inferior temporal gyrus L	Thalamus L
Cuneus R	Temporal pole: superior temporal gyrus L	Putamen R
Temporal pole: middle temporal gyrus R	Precentral gyrus L	Putamen L
Precentral gyrus L	Inferior frontal gyrus, triangular partR	Temporal pole: superior temporal gyrus L
Middle frontal gyrus, orbital part L	Cuneus L	Caudate L
Middle frontal gyrus, orbital part R	Temporal pole: middle temporal gyrus L	Precuneus R
Temporal pole: middle temporal gyrus L	Middle frontal gyrus, orbital part R	Precentral gyrus L
Inferior frontal gyrus, triangular part R	Thalamus R	Pallidum L

All the brain regions are from AAL (automated anatomical labelling).

R, right; L, left; SVM, support vector machine; LR, logistic regression; DL, deep learning.

### Exploring cross-site generalizability using leave-one-site-out cross-validation

In the present investigation, the cross-validation was carried out within each site and the average accuracy corresponded to the mean accuracy across all five sites. The reason for this approach is that, since different sites employed different recruitment criteria, different scanners and different scanning parameters, we expected site-related differences to be significant and larger than the differences between patients and controls. For completeness, however, we also used leave-one-site-out cross-validation, which involved training an algorithm based on four datasets and then testing it based on the remaining dataset; as expected, the accuracy using this alternative approach was lower than the accuracies for each individual site (50% for whole-brain images, 62.54–68.1% for functional connectivity, and 55–64.85% for graph-based metrics).

## Discussion

In the present article, we aimed to elucidate the neural correlates of schizophrenia by examining the relative diagnostic value of pre-processed whole-brain images, connectome-wide functional connectivity and graph-based metrics. Consistent with our first hypothesis, measures of functional integration (i.e. connectome-wide functional connectivity and graph-based metrics) allowed diagnostic classification with higher levels of accuracy than pre-processed whole-brain images. Critically, whole-brain images differ from connectome-wide functional connectivity and graph-based metrics in that they do not contain temporal information (i.e. functional connectivity). This pattern of results, therefore, provides support for the emerging notion that functional connectivity based on resting-state functional neuroimaging data, can be a powerful tool for characterizing brain disorders at the level of the individual (Camchong *et al*., 2011; Lei *et al*., 2015; Suo *et al*., 2015). For instance, Wee *et al*. (2012) used this approach to classify patients with amnestic mild cognitive impairment and healthy controls, achieving an accuracy of 86%. More recently, Iidaka and colleagues demonstrated similar results in autism spectrum disorder, by developing an algorithm that could classify patients and controls with approximately 90% accuracy (Iidaka, 2015). With respect to schizophrenia, Skatun *et al*. (2017) were able to discriminate between 182 patients with a schizophrenia spectrum diagnosis and 348 healthy controls with an accuracy of up to 80%, by using a whole-brain data-driven definition of network nodes and regularized partial correlations which revealed differences in functional connectivity within frontal, sensory, and subcortical networks between groups. In addition, Cheng *et al*. (2015), using measures of betweenness centrality extracted from resting-state functional MRI data, were able to classify 19 schizophrenic patients and 29 non-psychiatric controls with an accuracy of around 80%. Here we expand the existing literature by demonstrating that, amongst measures of functional integration, connectome-wide functional connectivity has greater diagnostic value than graph-based metrics, consistent with our second hypothesis. Nevertheless, the average diagnostic accuracy of connectome-wide functional connectivity was not very high, at around 81%. A possible explanation is that this might be due to the limited sample size of our datasets compared to some of the previous studies; e.g. Iidaka (2015) used rs-fMRI data to compare 312 patients with autism spectrum disorder and 328 controls with typical development. However, previous studies using a similar or smaller number of participants have reported comparable or even higher accuracies, suggesting that sample size may not be the only explanation. For example Cheng and colleagues used a total sample of 48 (19 patients, 29 controls) and reported accuracies up to 80% (Cheng *et al*., 2015; Khazaee *et al*., 2015); whereas Khazaee used 40 subjects (20 patients, 20 controls) and reported accuracies up to 100% (Cheng *et al*., 2015; Khazaee *et al*., 2015). An alternative explanation is that our average diagnostic accuracy of about 81% is an accurate reflection of the limited diagnostic value of connectome-wide functional connectivity when patients with a diverse range of symptoms and different durations of illness are combined into a single diagnostic group. A further source of heterogeneity is the type, dose, and duration of antipsychotic medications in four of the five datasets. We suggest that future studies might achieve higher diagnostic and prognostic accuracies by focusing on sub-groups of patients with similar clinical presentations; this, however, would require larger sample sizes than those used in the present investigation.

Graph-based theoretical analysis is thought to provide a powerful framework for characterizing topological properties of brain networks (Bullmore and Sporns, 2009; Biswal *et al*., 2010). Because many of these topological properties, including small-worldness, efficiency and nodal degree, have been shown to be altered in patients with schizophrenia relative to healthy controls (Fornito *et al*., 2012; Drakesmith *et al*., 2015), one might expect this information to have high diagnostic value at the level of the individual. However, in the present investigation, the use of graph-based metrics led to average accuracies that were just about higher than chance level (69%). This suggests that, while the extraction of graph-based analytic metrics from whole-brain time series reveals some important functional features at the group level, important information about network-level functioning at the individual level is lost during the computing. An alternative explanation is that our methodological approach, in which all graphic metrics were considered as simple vectors and were treated equally, was not optimal for the investigation of the diagnostic value of this specific feature. Future studies could examine the impact of using an alternative methodological approach, in which the different graphic metrics are associated with different *a priori* weights; here the main challenge would be to decide which *a priori* weights should be assigned to which graphic metrics which is unclear from the existing literature.

Consistent with our third hypothesis, our findings were consistent across different ML algorithms associated with varying degree of complexity and abstraction, including logistic regression, SVM and deep neural networks. This replicates a previous large-scale investigation (Sabuncu *et al*., 2015) reporting that the choice of measurement type used for classification has a much larger effect on the final accuracy than the choice of the ML algorithm. Previous studies have suggested that DL – a type of ML capable of high orders of complexity and abstraction (LeCun *et al*., 2015) which has already produced several promising results in medical image analysis (Kim *et al*., 2016; Havaei *et al*., 2017) – may yield higher classifier accuracy than SVM (Pinaya *et al*., 2016; Vieira *et al*., 2017). However, in the present study, DL did not perform better than shallow ML methods such as SVM or even LR despite significant differences in computing time and required computational resources. This might be explained by the relatively small sample size in each site, as DL models involve the estimation of a higher number of parameters that in turn require the use of a larger sample size.

Taken collectively, the results of the present investigation are consistent with the so-called ‘dysconnectivity hypothesis’ of schizophrenia, which attempts to elucidate the link between the symptoms of the illness with the underlying molecular and neuronal pathophysiology. Specifically, this hypothesis suggests that schizophrenia is best understood in terms of system-level aberrant neuromodulation of synaptic efficacy that mediates the influence of intrinsic and extrinsic connectivity (Friston *et al*., 2016). This aberrant neuromodulation is not equally distributed across the brain but is thought to affect certain neural circuitries more than others (Pettersson-Yeo *et al*., 2011). Here we found that the thalamus and temporal regions were amongst the areas providing the greatest contribution to classification according to all three ML methods. Alterations in thalamic functional connectivity are well-known features of psychotic disorders (Woodward and Heckers, 2016), and include both reduced prefrontal-thalamic connectivity and increased sensorimotor-thalamic (Woodward *et al*., 2012). These alterations are also evident in individuals at clinical high risk for schizophrenia, especially those who later go on to convert to psychosis (Anticevic *et al*., 2015), suggesting that they may represent a marker of future risk. Alterations in temporal connectivity have also been reported in several previous studies of people with psychosis as well as their unaffected relatives (Xiao *et al*., 2017). The thalamus and temporal regions have also been reported to show abnormal (i.e. increased) functional connectivity in patients with schizophrenia (Cetin *et al*., 2014). Our investigation extends these findings, which were based on group-level statistics, by suggesting that thalamic and temporal functional dysconnectivity is key for differentiating patients and controls at the level of the individual patients.

The results of the present study are unlikely to be explained by the differing effects of age or gender on brain function in patients and controls, as variation due to these factors was removed before estimating disease-related effects. Nevertheless, the present study has a number of limitations. First, our data were acquired at five different sites using different scanners and acquisition parameters; on the other hand, the use of independent datasets allowed us to demonstrate the replicability of our findings. Second, the available clinical information varied between the different datasets due to the use of different research protocols between sites. This means it was not possible to examine the relationship between clinically relevant factors and neural alterations across the five datasets. Third, in the present study, we did not use feature selection which has been shown to improve performance in previous studies (Mwangi *et al*., 2014); future studies could try to evaluate the impact of alternative feature selection strategies on the results. Fourth, the graph theoretical analysis was implemented using a widely popular approach based on Pearson's correlations. However, there are alternative approaches, such as partial correlation matrices and binary topology metrics, which could be considered in future studies. Fifth, the development of diagnostic biomarkers for schizophrenia has limited clinical utility in real-words clinical practice (Orrù *et al*., 2012). Of much greater clinical utility would be the development of biomarkers for predicting the onset of the illness or response to treatment; this, however, will require the availability of follow-up clinical data which were not available in our groups.

In conclusion, despite the above limitations, the present study demonstrates that connectome-wide functional connectivity allows the identification of individual patients with schizophrenia with greater accuracy than pre-processed whole-brain images and graph-based metrics. Functional dysconnectivity of thalamic and temporal regions was key for differentiating patients and controls at the level of the individual patients. This pattern of results is consistent with the dysconnectivity hypothesis of schizophrenia, which states that the neural basis of the disorder is best understood in terms of system-level functional connectivity alterations. Future studies could investigate the extent to which this finding is specific to schizophrenia or a trans-diagnostic feature of psychiatric disorders.

Finally, it is important to acknowledge that, at present, neuroimaging is still far from becoming a useful tool in the day-to-day clinical practice of clinical psychiatry. One of the key challenges is the poor generalizability of the findings across different datasets. For example, when using leave-one-site-out cross-validation, we found poor generalizability across the five sites, possibly due to the use of different recruitment criteria, scanners and scanning parameters. Nevertheless, the present study enhances the current understanding of network-level abnormalities in psychosis, which in turn could inform the development of diagnostic and prognostic neuroimaging-based markers in the future.

## Supplementary material

For supplementary material accompanying this paper visit https://doi.org/10.1017/S0033291719001934. click here to view supplementary material
