# Supplementary material for: Detecting schizophrenia at the level of the individual: relative diagnostic value of whole-brain images, connectome-wide functional connectivity and graph-based metrics
Source: Psychol Med. 2019 Aug 8;50(11):1852–61. doi: 10.1017/S0033291719001934 (PMC7477363; doi:10.1017/S0033291719001934)
Supplement: Supplementary file 1 [file S0033291719001934sup.zip › S0033291719001934sup003.docx]

**Supplemental Information**

**Participants**

Dataset 1 was obtained from the Neuroimaging Informatics Tools and Resources Clearinghouse (NITRC) website and was provided by the Centers of Biomedical Research Excellence (COBRE; <http://fcon_1000.projects.nitrc.org/indi/retro/> cobre.html). In this dataset, a diagnosis of schizophrenia was made using the Structured Clinical Interview for DSM Disorders (SCID; Diagnostic and Statistical Manual of Mental Disorders, DSM-IV) (First *et al.*, 2012). Exclusion criteria included confirmed or suspected pregnancy, any history of neurological disorders and a history of intellectual disability. The patients were all receiving various antipsychotic medications at the time of the study (no medication changes in 1 month). Written informed consent was obtained from participants according to institutional guidelines at the University of New Mexico.

Dataset 2, acquired as part of the UCLA Consortium for Neuropsychiatric Phenomics LA5c Study, was obtained from the OpenfMRI database (accession number: ds000030). All patients underwent a semi-structured assessment with the Structured Clinical Interview for the Diagnostic and Statistical Manual of Mental Disorders, Fourth Edition (DSM-IV) (First *et al.*, 2004). Exclusion criteria included left-handedness, pregnancy, history of head injury with loss of consciousness or cognitive sequelae, or other contraindications to scanning. Stable medications were permitted for the patients and 81% of patients had at least one comorbid diagnosis (Poldrack *et al.*, 2016). After receiving a verbal explanation of the study, participants gave written informed consent following procedures approved by the Institutional Review Boards at UCLA and the Los Angeles County Department of Mental Health.

Dataset 3 was acquired at Maastricht University, The Netherlands. Patients were recruited through clinicians working in selected representative geographic areas in the Netherlands and Belgium. Diagnosis of schizophrenia was based on DSM-IV criteria (Association, 2000), assessed with the Comprehensive Assessment of Symptoms and History (CASH) interview (Andreasen *et al.*, 1992). Exclusion criteria included confirmed or suspected pregnancy, any history of neurological disorders, a history of intellectual disability and/or a history of substance abuse/dependence within the last 12 months. Antipsychotic medication use for patients was determined using the reports of the participant’s psychiatrist. The ethics committee of Maastricht University approved the study, and all the participants gave written informed consent in accordance with the committee’s guidelines and the Declaration of Helsinki.

Dataset 4 was acquired in Dublin and scanned at the Trinity College Institute of Neuroscience as part of a Science Foundation Ireland-funded neuroimaging genetics study ("A structural and functional MRI investigation of genetics, cognition and emotion in schizophrenia"). Patients with confirmed DSM-IV diagnosis of schizophrenia were recruited through local clinical services. Exclusion criteria included confirmed or suspected pregnancy, any history of neurological disorders or intellectual disability and substance misuse in the preceding three months. All patients were chronic, but stable, medicated outpatients, with a confirmed diagnosis. Participants provided written, informed consent in accordance with local ethics committee guidelines.

Dataset 5 was acquired at the West China Hospital of Sichuan University, Chengdu, China. An initial diagnosis of schizophrenia and duration of illness were determined by consensus between 2 experienced psychiatrists, using the Structured Clinical Interview for DSM-IV(SCID)-Patient Version (Association, 2000). In addition, diagnosis of schizophrenia was confirmed for all the patients at 1-year follow-up. Exclusion criteria were the existence of a neurological disorder or other psychiatric disorders, alcohol or drug abuse (DSM-IV), pregnancy, and any chronic physical illness such as a brain tumor, hepatitis, or epilepsy, as assessed by clinical evaluations and medical records. All patients are drug-naive first-episode schizophrenia. The study was approved by the ethics committee of West China Hospital, and written informed consent was obtained from all participants.

**MRI acquisition**

At each site the rs-fMRI images were acquired by the Echo-Planar Imaging (EPI) sequence. Dataset 1 was acquired using a 3T Siemens scanner. The sequence parameters were as follows: repetition time/echo time (TR/TE) = 2000/30 ms; flip angle = 90°; 33 axial slices per volume; voxel size = 3.75 × 3.75 × 4.55 mm3; number of volumes= 150. Dataset 2 was acquired using a 3T Siemens scanner. The sequence parameters were as follows: repetition time/echo time (TR/TE) = 2000/30 ms; flip angle = 90°; 34 axial slices per volume; voxel size = 3 × 3 × 4 mm3; number of volumes= 152. Dataset 3 was acquired using a 3T Siemens Magnetom Allegra head scanner. The sequence parameters were as follows: repetition time/echo time (TR/TE) = 1500/30 ms; flip angle = 90°; 27 axial slices per volume; voxel size = 3.5 × 3.5 × 5.2 mm3; number of volumes= 200. Dataset 4 was acquired using a 3T Philips Intera Achieva scanner. The sequence parameters were as follows: repetition time/echo time (TR/TE) = 2000/30 ms; flip angle = 90°; 35 axial slices per volume; voxel size = 3.5 × 3.5 × 3.5 mm3; number of volumes= 180. Dataset 5 was acquired using a 3T GE scanner (EXCITE; General Electric, Milwaukee, Wisconsin). The sequence parameters were as follows: repetition time/echo time (TR/TE) = 2000/30 ms; flip angle = 90°; 30 axial slices per volume; voxel size = 3.75 × 3.5 × 5 mm3; number of volumes= 200.

**Dealing with potential motion-related artifacts.**

To deal with potential artefacts arising from motion or other factors, we applied Friston 24-parameter correction colleagues (Yan *et al.*, 2013). In addition, to ensure that motion artifacts were not contributing to the observed group differences, we employed the “head motion scrubbing” method proposed by Power and colleagues (Power *et al.*, 2014).

**Machine learning models.**

In the present study, we used three machine learning methods - logistic regression (LR), support vector machine (SVM) and deep learning (DL) to perform single-subject classification. These methods were chosen in light of their widespread use amongst the neuroimaging community and their varying degrees of complexity and abstraction. In particular, LR is considered to be the “simplest” ML method; SVM is associated with medium levels of complexity and abstraction, and is the most widely used ML method in psychiatric neuroimaging; and DL is thought to have the greatest potential of detecting hidden patterns in the data, due to its higher levels of complexity and abstraction. These methods are described in detail below.

*Treatment of whole brain images.* For LR and DL, the whole brain images were treated as 3D * time data. Here we treated each time point separately and computed the subject's final prediction based on the average probability across all time points; for SVM, the whole brain images were treated as 4D data. The different treatment of whole brain images was due to computational constrains (i.e. memory limitation and computing time) affecting the implementation of LR and DL but not SVM.

*Logistic Regression.* The LR model fits a separating hyperplane, i.e. a linear function of the input features, between different classes (e.g. patients and controls). Within this framework, the probability that an observation belongs to a specific class is determined by the sigmoid function of a weighted sum of the input. These weights are estimated using a training sample and then assessed using an independent testing sample. In the present study, logistic regression was implemented using the Lasagne library (<https://zenodo.org/record/27878>). We initialized the weights of the classifier using the Glorot method (Glorot and Bengio, 2010), with parameters sampled from the uniform distribution, and initialized the bias with zero value. These parameters were then optimized using the Rmsprop algorithm (Tieleman and Hinton, 2012) using a mini-batch with 16 training samples, 100 training epochs, and a learning rate starting with a value of 0.05 with a learning rate decay decrease a factor of 10-6 per epoch. An L2 penalization with parameter value equal to 5*10‑5 was applied to the model to penalize weights with excessively high values and to prevent overfitting.

*Support Vector Machine.* The SVM model (Cortes and Vapnik, 1995) maps the input vectors to a feature space using a set of mathematical functions known as kernels. In this feature space, the model finds the optimum separation surface that can maximise the margin between different classes within a training dataset. Once the separation surface is determined, it can be used to predict the class of new observations using an independent testing dataset. Here a linear kernel was preferred to a nonlinear one to minimise the risk of overfitting. The model was based on libsvm and implemented using the Scikit-Learn library (Pedregosa *et al.*, 2012). The linear SVM has only one parameter (soft margin parameter C) that controls the trade-off between reducing training errors and having a larger separation margin. This parameter was optimized spitting the data into training and validation sets and performing a grid search (i.e., C = 2-5, 2-3, 2-1, 2-0, 21, 23 … 213, 215) to estimate the best value.

*Deep Learning Technology.* DL models are a set of machine learning algorithms that extract multiple levels features from the input data (Vieira *et al.*, 2017). Here higher-level features are learned as a non-linear combination of lower-level features, allowing the extraction of complex and abstract patterns in the data. Based on these higher-level features, the model determines the separation surface to perform the classification task. Here we used two types of deep learning models: the Convolutional Neural Network (ConvNet) and a deep neural network with fully connected layers.

Because whole brain images data are associated with high dimensionality (over 50,000 voxels), the application of a deep neural network with fully connected layers to this type of data would require excessive computational resources. However, since whole brain time series data are also associated with correlation between neighbouring voxels, we used ConvNet (where the units are not connected to all the units in the previous layer) to reduce the number of parameters in the network. This reduction had the impact of reducing risk of overfitting whilst alleviating computational demands. The training of the ConvNet was performed using 3D kernels in which each time step was considered an input channel; training was implemented using the Rmsprop algorithm with a mini-batch with 16 training samples, 100 training epochs and a learning rate starting with a value of 0.05 and decaying a factor of 10-6 per epoch. The batch normalization (Ioffe and Szegedy, 2015) was applied to all layers to accelerate the convergence of the training. The architecture of the deep learning model is represented in Figure S1.

In contrast, it was possible to use a deep neural network with fully connected layers to analyse connectome-wide matrices and graph-based analytic metrics, in light of the lower dimensionality of these measures. In particular, we adopted a structure with three hidden layers with 100 units in each hidden layer. We used the Rectified Linear Unit (ReLU) in all hidden layers and the softmax units in the output layer. The training of the network was performed using the Rmsprop algorithm using a mini-batch with 16 training samples, 100 training epochs, and a learning rate starting with a value of 0.05 with a learning rate decay decrease a factor of 10-6 per epoch. The L2 parameter was set to 5*10-5. Both deep learning models were implemented using the Lasagne library.

References

**Andreasen, N. C., Flaum, M. & Arndt, S.** (1992). The Comprehensive Assessment of Symptoms and History (CASH). An instrument for assessing diagnosis and psychopathology. *Archives of General Psychiatry* **49**, 615-23.

**Association, A. P.** (2000). Diagnostic and Statistical Manual of Mental Disorders fourth edition, Text revision (DSM-IV TR).

**Cortes, C. & Vapnik, V.** (1995). Support Vector Network. *Machine Learning* **20**, 273-297.

**First, M. B., Frances, A. & Pincus, H. A.** (2004). *DSM-IV-TR guidebook*. American Psychiatric Pub.

**First, M. B., Spitzer, R. L., Gibbon, M., Williams, J. B. W., Spitzer, R., Gibbons, M. & Williams, J.** (2012). *Structured Clinical Interview for DSM-IV Axis I Disorders (SCID-I/P)*. John Wiley & Sons, Inc.

**Glorot, X. & Bengio, Y.** (2010). Understanding the difficulty of training deep feedforward neural networks. *Journal of Machine Learning Research* **9**, 249-256.

**Ioffe, S. & Szegedy, C.** (2015). Batch Normalization: Accelerating Deep Network Training by Reducing Internal Covariate Shift. *Computer Science*.

**Pedregosa, F., Varoquaux, G., Gramfort, A., Michel, V., Thirion, B., Grisel, O., Blondel, M., Prettenhofer, P., Weiss, R. & Dubourg, V.** (2012). Scikit-learn: Machine Learning in Python. *Journal of Machine Learning Research* **12**, 2825-2830.

**Poldrack, R. A., Congdon, E., Triplett, W., Gorgolewski, K. J., Karlsgodt, K. H., Mumford, J. A., Sabb, F. W., Freimer, N. B., London, E. D., Cannon, T. D. & Bilder, R. M.** (2016). A phenome-wide examination of neural and cognitive function. *Scientific Data* **3**, 160110.

**Power, J. D., Mitra, A., Laumann, T. O., Snyder, A. Z., Schlaggar, B. L. & Petersen, S. E.** (2014). Methods to detect, characterize, and remove motion artifact in resting state fMRI. *Neuroimage* **84**, 320-41.

**Tieleman, T. & Hinton, G.** (2012). Lecture 6.5-rmsprop: Divide the gradient by a running average of its recent magnitude. *COURSERA: Neural networks for machine learning* **4**, 26-31.

**Vieira, S., Pinaya, W. H. & Mechelli, A.** (2017). Using deep learning to investigate the neuroimaging correlates of psychiatric and neurological disorders: Methods and applications. *Neuroscience and Biobehavioral Reviews* **74**, 58-75.

**Yan, C. G., Craddock, R. C., He, Y. & Milham, M. P.** (2013). Addressing head motion dependencies for small-world topologies in functional connectomics. *Frontiers in Human Neuroscience* **7**, 910.

Figure legends

**Figure S1.  The architecture of the deep learning model.**

We used a convolutional neural network in which the first layer was a 3D convolutional layer with 64 filters (each filter with size 7x7x7 voxels), a stride with size of 2, and "valid" padding (V). Next, a max pooling layer was used to reduce the dimensionality of the feature maps of the network. This layer had filter sizes of 5x5x5 voxels and stride size of 3. The third layer was a convolutional layer with 64 filters (each filter with size 5x5x5 voxels), a stride size 1, and a "same" padding. Next, we used a max pooling layer with filter size of 5x5x5 voxels and a stride size of 3 voxels. Finally, the feature maps were flattened from a 3D space to a 1D vector in order to be used as input to the next layer: a full-connected (FC) layer. This layer had 50 rectified linear units (ReLU). Lastly, the output layer had 2 units with softmax activation function that outputs the probability of the input data being from a patient or control.
