# Supplementary figures and images for: Detecting schizophrenia at the level of the individual: relative diagnostic value of whole-brain images, connectome-wide functional connectivity and graph-based metrics
Source: Psychol Med. 2019 Aug 8;50(11):1852–61. doi: 10.1017/S0033291719001934 (PMC7477363; doi:10.1017/S0033291719001934)

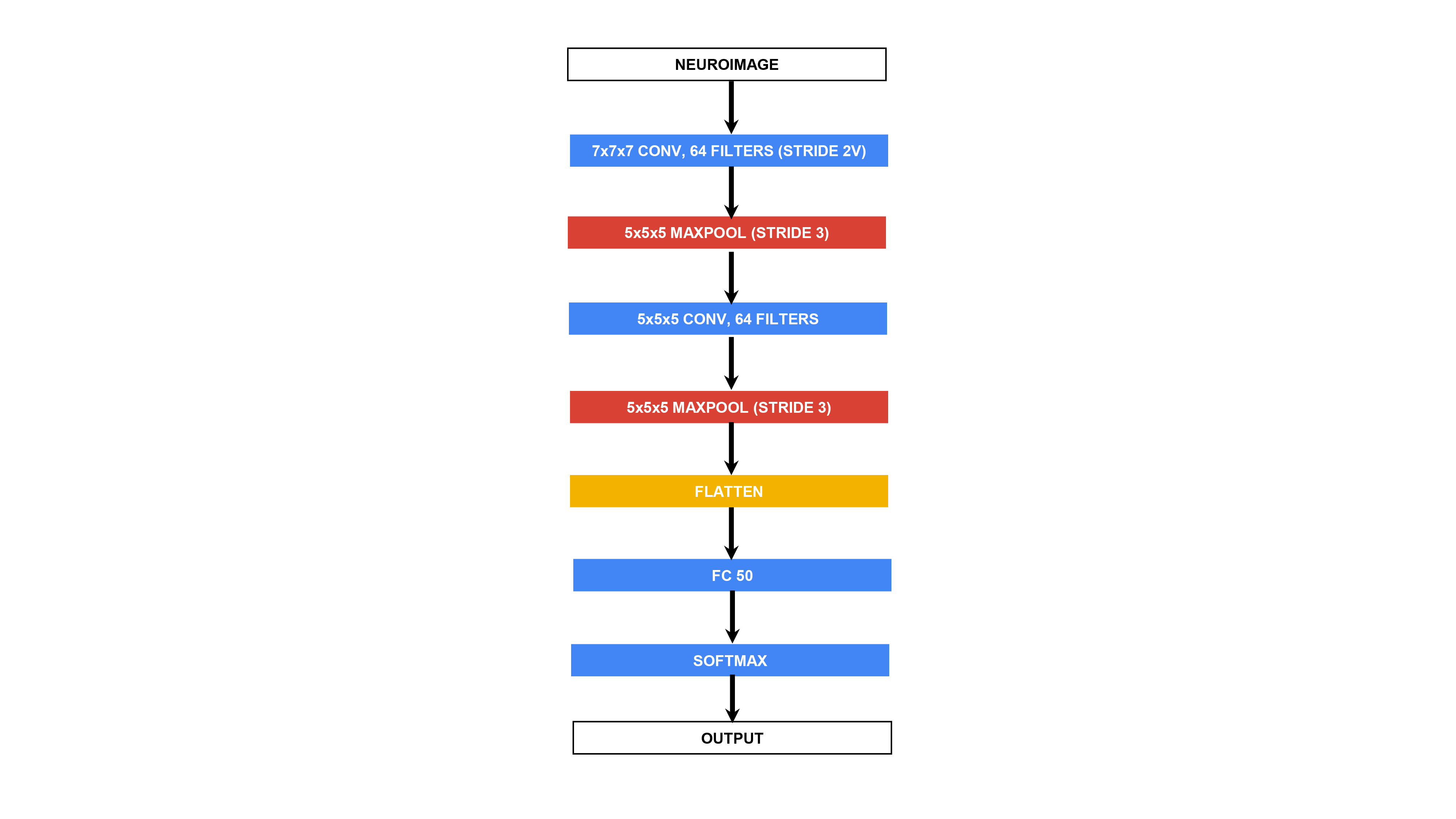

Supplement: Supplementary file 1 [file S0033291719001934sup.zip › S0033291719001934sup001.tif]

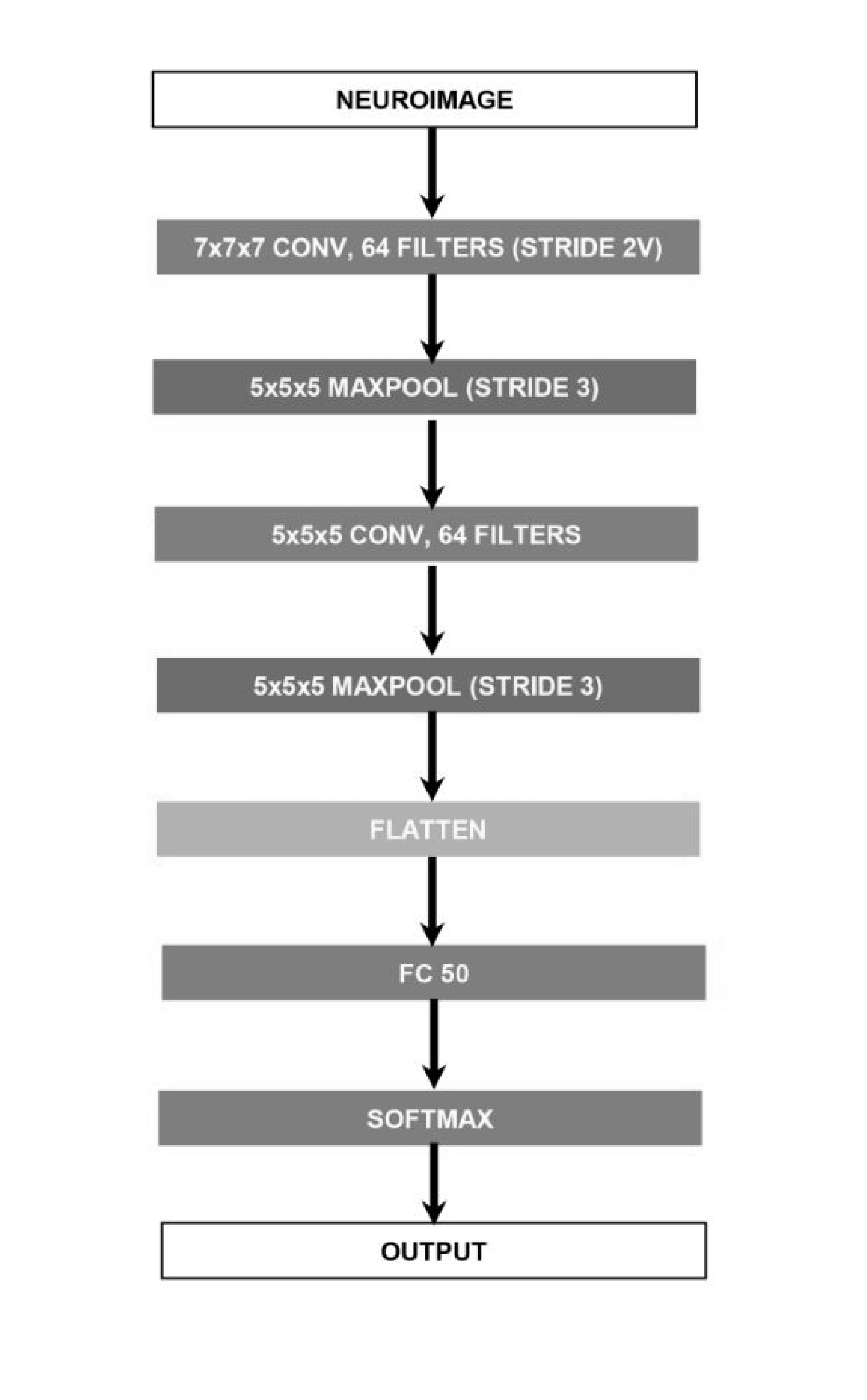

Supplement: Supplementary file 1 [file S0033291719001934sup.zip › S0033291719001934sup002.tif]
